# The aggregate and sectoral time-varying market efficiency during crisis periods in Turkey: a comparative analysis with COVID-19 outbreak and the global financial crisis

**DOI:** 10.1186/s40854-023-00484-4

**Published:** 2023-05-01

**Authors:** Deniz Erer, Elif Erer, Selim Güngör

**Affiliations:** 1 School of Applied Sciences, Manisa Celal Bayar University, Manisa, Turkey; 2 Independent Researcher, Izmir, Turkey; 3 Resadiye Vocational School, Tokat Gaziosmanpasa University, Tokat, Turkey

**Keywords:** MF-DFA, Adaptive market hypothesis, Global financial crisis, COVID-19 outbreak, Sectoral indices, C58, G14, E44

## Abstract

This study aims to examine the time-varying efficiency of the Turkish stock market’s major stock index and eight sectoral indices, including the industrial, financial, service, information technology, basic metals, tourism, real estate investment, and chemical petrol plastic, during the COVID-19 outbreak and the global financial crisis (GFC) within the framework of the adaptive market hypothesis. This study employs multifractal detrended fluctuation analysis to illustrate these sectors’ multifractality and short- and long-term dependence. The results show that all sectoral returns have greater persistence during the COVID-19 outbreak than during the GFC. Second, the real estate and information technology industries had the lowest levels of efficiency during the GFC and the COVID-19 outbreak. Lastly, the fat-tailed distribution has a greater effect on multifractality in these industries. Our results validate the conclusions of the adaptive market hypothesis, according to which arbitrage opportunities vary over time, and contribute to policy formulation for future outbreak-induced economic crises.

## Introduction

Financial crises are one of the primary causes of financial asset depreciation. For example, the global financial crisis (GFC), which began in the United States’ housing market, first affected financial markets and then the real economy via financial channels (Kristoufek 2012; Bernanke 2018). The aforementioned crisis has impacted the financial markets and real economies of developing countries, particularly Turkey.

The COVID-19 outbreak caused another crisis that significantly impacted the values of financial assets. Because of the increased uncertainty in the economy, the authorities’ measures to control the spread of the COVID-19 outbreak have significantly impacted financial markets. According to Ozkan (2021), the COVID-19 outbreak has caused a more severe and sudden deterioration in the global economy than the GFC. The reason for this is that the COVID-19 outbreak has brought the economy to a halt by contemporaneously destroying demand and supply chains as a result of the extensive lockdowns. Thus, financial investors must make a decision that provides the maximum return with minimum risk.

The efficient market hypothesis (EMH) proposed by Fama (1970) constitutes a substantial theoretical framework for the aforementioned subject. According to this hypothesis, financial asset prices represent all available information. Current prices of the financial assets include all past information involved in price changes, implying that the market has weak efficiency (Fama 1970). The effect of information on the price formation may be unclear due to the vague information flow. In such a case, the investors’ actions significantly reduce the possibility of establishing arbitrage circumstances. Because prices with a random walk cannot be predicted in an efficient market, an asset price cannot be predicted based on its historical price. However, asset prices can represent the effect of new information if a market is inefficient (Pagan 1996; Mensi et al. 2017; Tiwari et al. 2019). In an efficient market, all investors are assumed to receive new information simultaneously and cannot profit excessively. Furthermore, systematic anomalies, such as herd behavior and the day of the week effect, do not exist, and asset prices are completely random. Thus, the validity of the EMH is frequently tested by examining whether asset prices follow a random walk process. Asset prices must follow a random walk process, resulting from the movement of many investors attempting to capitalize on the opportunity of the received information for the EMH to be valid. Many studies (e.g., Lee 1992; Elliott et al. 1996; Mehmood et al. 2012; Valera and Lee 2016; Mensi et al. 2017; Lee et al. 2018; Tiwari et al. 2019; Dias et al. 2020) have found that prices pursue a random walk by investigating the foreseeability of asset returns based on past price movements.

The EMH can be classified into three groups: *weak-form*, *semi-strong form*, and *strong form*. The weak form of market efficiency indicates that investors cannot obtain abnormal returns from past asset price movements because market prices have already reflected all past and current information about price and volume. Meanwhile, the semi-strong form of market efficiency states that the market value of an asset adapts instantly to all new information in pursuit of a random walk process from past prices. Lastly, the strong-form market efficiency hypothesis holds that a single investor cannot make abnormal profits even with insider information (Malkiel 2003; Cagli 2018). In general, the EMH has an “*all-or-none*” restriction (Lim and Brooks 2011). Some studies on behavioral finance (Hirshleifer 2002; Ritter 2003; Ghazani and Araghi 2014; Hiremath and Narayan 2016; Tuyon and Ahmad 2016; Kapoor and Prosad 2017; Al-Khazali and Mirzaei 2017; Okorie and Lin 2021) have considered that market efficiency within the “*all-or-none*” scope is limited because the inclusion of information in the market conditions is a complicated and non-instant process. Starting with behavioral finance, Lo (2004) suggested the adaptive market hypothesis (AMH) to account for both calendar anomalies and the EMH. In terms of evolutionary principles, the AMH associates market efficiency with behavioral choices. According to this theory, return predictability might vary over time due to market conditions such as crises, turmoil, and bubbles. In the AMH, market efficiency is determined by a qualification that varies over time and across the market but is essentially an “*all-or-none*” restriction. Consequently, market efficiency may change cyclically rather than following a trend against the higher efficiency assumed by the EMH (Choi 2021; Al-Khazali and Mirzaei 2017; Rodriguez, et al. 2014; Tuyon and Ahmad 2016).

Various events have varying degrees of influence on stock market efficiency. Events that cause widespread panic and instability, such as exchange rate shocks, economic and financial crises, bubbles, and pandemics, frequently worsen the EMH because they cause financial asset prices to diverge from their fundamental values (Kim et al. 2011; Niemczak and Smith 2013; Rodriguez et al. 2014; Charfeddine and Khediri 2016; Rahman et al. 2017; Lalwani and Meshram 2020; Ozkan 2021). Within this scope, this study aims to analyze the aggregate and sectoral market efficiency in the context of the AMH during the GFC and the COVID-19 outbreak.

To this end, the following issues will be focused in this research:

- How does the COVID-19 outbreak affect aggregate market efficiency in the framework of AMH?
- Does the effect of the COVID-19 outbreak on market efficiency vary depending on the AMH sub-sectors?
- Which sectors’ efficiency is the most eroded?
- Is the sectoral market efficiency differentiated for the GFC and COVID-19 outbreak periods?

This study is expected to contribute to the market efficiency literature in several ways: First, we investigate the market efficiency with a time-varying degree on an aggregate and sectoral basis for two different crisis periods: the COVID-19 outbreak and the GFC. Because the COVID-19 outbreak affects the economy differently at each sectoral level, investors want to forecast future sectoral returns. Hence, we analyze how the COVID-19 outbreak differs from the GFC in terms of economic effect. Time-varying techniques aid in understanding the market properties associated with structural revolutions. Second, we employ the multifractal detrended fluctuation analysis (MF-DFA) model to assess and compare aggregate and sectoral market efficiency during times of crisis. Kantelhardt et al. (2002) proposed this method to analyze the multifractal non-stationary time series and determine long-range and short-range dependence. Therefore, financial investors must examine the efficiency of each sector to determine their investment strategy. Third, we clarify aggregate and sectoral market efficiency in the AMH framework for various crisis periods.

There are numerous studies in the literature that investigate the market efficiency of Borsa Istanbul. Some studies (Smith and Ryoo 2003; Ozdemir 2008; Karan and Kapusuzoglu 2010; Gozbasi et al. 2014; Cagli 2018; Özdemir 2022) have shown that the Turkish stock market is weak-form efficient, whereas others (Özer and Ertokatli 2010; Aliyev 2019; Hailu and Vural 2020; Altuntaş et al. 2022) suggest that the EMH is valid for Borsa Istanbul. Furthermore, Bozkuş and Kahyaoğlu (2020) demonstrated the validity of the heterogeneous market hypothesis, whereas Balcı et al. (2022) demonstrated the validity of the fractal market hypothesis in Borsa Istanbul. Meanwhile, Mandacı et al. (2019), Eyüboğlu and Eyüboğlu (2020), and Özkan (2020) and Burhan and Acar (2021) discovered that AMH holds true in the Turkish stock market. Mensi et al. (2022) also examined the asymmetric multifractal structure of the markets during various crisis periods, such as the GFC and COVID-19, using the leading market indices of the Middle East and North Africa (MENA) stock markets, including Turkey. They obtained various evidence on a country basis. However, we only observe one study (Choi 2021) on the effect of the GFC and COVID-19 outbreak on sectoral market efficiency in the United States. Unlike Choi’s (2021) study, we intend to investigate sectoral market efficiency in the Turkish stock market during the GFC and COVID-19 outbreak periods. As far as we know, this is the first study to examine the effects of the COVID-19 outbreak and the GFC on aggregate and sectoral market efficiency in Turkey using MF-DFA. Therefore, our findings are expected to contribute to policy formulation for future turbulent periods induced by outbreaks and financial crises.

The following sections are planned: “Literature review” Section summarizes literature review on market efficiency. “Data” Section explains the data. “Methodology” Section provides context for the methodology. “Empirical findings” Section demonstrates the empirical findings. Finally, “Conclusion” Section concludes by discussing the results and making recommendations.

## Literature review

In finance, the assumption of market efficiency is the basis of each model, strategy, and policy in stock markets. Since its inception in the 1960s, the concept of market efficiency has been the subject of intense empirical and theoretical debate, based on several hypotheses like the random walk hypothesis, the EMH, and the AMH. The EMH has been an important area of research to understand better and promote the quality of stock markets; as a result, numerous studies have been conducted to provide a better understanding and testing of the EMH. Lim et al. (2008) investigated weak-form market efficiency for ten Asian emerging economies using linear and nonlinear tests. The results show that political and economic events can explain each economy’s market efficiency; however, cross-country differences in market efficiency can be attributed to trading activity and market size. Meanwhile, Pele and Voineagu (2008) analyzed the market efficiency in the Romanian stock exchange using the ARIMA models and discover that the market exhibits weak efficiency. For the Australian stock market, Worthington and Higgs (2009) investigated efficiency in the weak form using linear unit root tests, coefficient of autocorrelation runs, and multiple variance ratio (MVR) tests. The autocorrelation coefficient results show that daily stock returns are inefficient, and monthly returns are only marginally efficient. Furthermore, the MVR test results indicate that the daily series do not exhibit weak-form efficiency, and the monthly series exhibit random walk behavior. In contrast, the run and unit root test results show that all series violated weak-form efficiency. Using autocorrelation, runs, and variance ratio (VR) tests, Loc et al. (2010) examined whether the Vietnamese stock market is efficient in the weak-form. They detected that the market is inefficient and in a weak state.

Rejichi and Aloui (2012) implemented the Hurst exponent approach to assess the efficiency of the MENA stock markets. They reported that Iran has the highest level of inefficiency in this region, whereas Israel, Turkey, and Egypt have the lowest levels of inefficiency. Rizvi et al. (2014) employed MF-DFA to assess stock market efficiency in Islamic and developed countries. They asserted that traditional countries are more efficient than Islamic markets, and that Islamic markets are highly efficient, especially during crisis periods. Meanwhile, Ito et al. (2014) used a non-Bayesian time-varying vector autoregressive model to argue the market efficiency of G7 countries and the time-varying structure of international connections. Their findings suggest that global connections and market efficiency vary over time, and that their behaviors are positively influenced by historical events involving the international financial system. Kumar (2014) utilized DFA and local Whittle methods to empirically investigate the validity of the EMH in India’s major industry indices. The findings demonstrate how dynamic the efficiency characteristics of Indian industrial indices are. Gozbasi et al. (2014) used ESTAR unit root tests to examine the EMH in the Turkish stock market and discover that Borsa Istanbul stock prices follow a random walk process and that the Turkish stock market has weak-form efficiency. Sensoy and Tabak (2015) used the generalized Hurst exponent approach to empirically demonstrate time-varying inefficiency in stock markets in the European Union. Their findings indicate that the Eurozone sovereign debt crisis only negatively impacts the markets of France, Spain, and Greece,, whereas the GFC negatively impacts nearly all stock markets. Arshad et al. (2016) employed the MF-DFA approach to examine the stock market efficiency of the Organization of Islamic Conference member countries. Their findings show that efficiency has increased over the last decade. Charfeddine and Khediri (2016) used generalized autoregressive conditional heteroskedasticity (GARCH) in mean and DFA techniques to test time-varying efficiency in the weak-form for the Gulf Cooperation Council stock markets and find that market efficiency varies during times of financial turbulence, such as the Arab Spring and the subprime crisis. Stakic et al. (2016) employed non-parametric and parametric tests to examine market efficiency in Serbia and discover that the market is inefficient in its weak form. Wen et al. (2019) analyzed the impact of retail investor attention on stock price crash risk in China. They found that retail investor attention negatively affects crash risk and affects information asymmetry. Meanwhile, Li et al. (2021) stated that financial series have complex structures due to human behavior and changing economic environments. They proposed a new approach, a revised SVDD model, to efficiently determine the number of clusters in financial data and concluded that this model detects financial series subpatterns successfully.

Regarding market efficiency, some studies used the MF-DFA approach to investigate multifractal characteristics of stock markets. Mensi et al. (2017) used the MF-DFA model to explore efficiency in the weak-form in Islamic sectoral stock markets. Their findings show that sectoral stock market efficiency changes over time, with the level of efficiency decreasing following the GFC. They also concluded that these markets are likely to exhibit high efficiency in the long run, while market efficiency is realized at a reasonable level in the short run. Rizvi and Arshad (2017) estimated Japanese stock market efficiency and integration over time using the multivariate GARCH and MF-DFA models. They claimed that market efficiency has increased over time, with each successive recession causing a decrease in integration levels. Al-Yahyaee et al. (2018) used the MF-DFA model to compare the efficiency of stock, bitcoin, gold, and exchange rate markets and discover that the Bitcoin market is less efficient than other markets due to its stronger multifractal structure. Ali et al. (2018) compared the efficiency of four Islamic stock markets to eight traditional stock markets using the MF-DFA approach. They demonstrated that the developed markets are more efficient than other markets. Furthermore, their results show that, except for Jordan, Pakistan, and Russia, almost all Islamic stock markets are more efficient than conventional country markets. Using MF-DFA and the Hurst exponent, Bouoiyour et al. (2018) argued whether the Islamic stock market is efficient. Their findings show that developed Islamic markets are more efficient than emerging ones.

Furthermore, their results show that the long-run (short-run) behavior of the emerging (developed) Islamic stock market is inconsistent, whereas the short-run (long-run) behavior of the developed Islamic stock market is consistent. Han et al. (2019) utilized the MF-DFA model to compare the efficiency of the Chinese stock market before and after the 2015 stock market turbulence and report that the returns for indices have multifractal features with different levels in the sample, resulting in stock market inefficiency. Tiwari et al. (2019) used the MF-DFA model to examine the efficiency and multifractality of eight developed and three emerging stock markets. Their findings show that stock markets are multifractal and generally consistent in the long run. They also claimed that most markets are inefficient in the short run but efficient in the long run. Miloş et al. (2020) estimated the multifractal characteristics of seven Central and Eastern European (CEE) stock markets using the MF-DFA approach and find that the CEE stock markets are not efficient. Choi (2021) used the MF-DFA approach to compare the efficiency of the US stock market during the COVID-19 and GFC periods. They stated that the returns exhibit both inconsistent and consistent characteristics during the GFC and the COVID-19 outbreak. They also exhibited that the sectors with the highest and lowest levels of efficiency in both crisis periods are public services and consumer discretionary. Mensi et al. (2022) utilized an asymmetric MF-DFA model to investigate the multifractality of 10 MENA stock markets during the GFC, oil price crash, and COVID-19 outbreak periods. Their results reveal that the Turkish stock market is the least ineffective among MENA stock markets in both uptrends and downtrends. Furthermore, their results show that, when compared to other periods, the period of the COVID-19 outbreak had the most insufficient markets.

The disagreement over whether the stock market is fully or adaptively efficient has increased academic interest in the AMH. Several studies have been conducted to investigate the effects of the AMH on stock markets. Ghazani and Araghi (2014) estimated the presence of AMH in the Tehran stock market using linear and nonlinear models. Smith and Dyakova (2014) utilized VR tests to investigate the efficiency of eight African stock markets and show that the AMH is valid in related stock markets. Rodriguez et al. (2014) used the DFA technique to argue the efficiency of the US stock market on different time scales and report that market efficiency varies across scales and is influenced by cyclical dynamics. They also discovered that annual time scales are more efficient than other time scales. Hiremath and Narayan (2016) applied the generalized Hurst exponent approach to test the AMH’s validity in the Indian stock market and discover that the Indian stock market has come a long way in terms of efficiency. They also detected a significant and positive link between the Indian market’s efficiency deficit, major domestic policy, financial crises, other international turbulences, and crisis-related cases. Ito et al. (2016) used the time-varying autoregressive model to assess the efficiency of the US stock market, revealing that the market is generally efficient except for the recession periods of 1873–1879 and 1902–1904, the New Deal period, and the 1957–1958 recession and post-recession period. Tuyon and Ahmad (2016) investigated the efficiency of the Malaysian stock market by implementing VR, ordinary least squares, and quantile regression tests. The efficiency tests reveal the adaptive weak market efficiency model’s trends across economic stages and market conditions. Al-Khazali and Mirzaei (2017) utilized the mean–variance and stochastic dominance models to analyze the existence of the AMH in eight Islamic stock indices using calendar anomalies. They determined that time-varying calendar anomalies in Islamic stock indices favor the AMH. Similarly, Okorie and Lin (2021) investigated the adaptive form of market efficiency in Brazil, India, Russia, and the US economies during the COVID-19 outbreak. Their results show no significant change in the Brazilian and US stock markets’ long, medium, or short run market efficiency levels. Furthermore, they ascertained that the Russian stock market is more information-efficient in the long run than the Indian stock market. Ozkan (2021) employed the wild bootstrap automatic VR and automatic portmanteau tests to examine the stock market efficiency of six developed economies, namely the United Kingdom, the United States, Spain, Italy, Germany, and France, which were all heavily affected by the COVID-19 outbreak. According to the results, deviations from market efficiency during the outbreak are greater in the UK and US than in other stock markets.

Mandelbrot (1971) and Peters (1991) showed that the properties of financial series characteristics such as fat-tailed and leptokurtic can lead to complexity, discontinuity, and nonlinearity. Therefore, Peters (1994) proposed the fractal markets hypothesis, which is based on Fractal Geometry, and claims that illiquidity causes extreme and unstable market movements. Mandelbrot (1997) found that fluctuations in financial series over different time segments reflect a fractal structure in nature. According to Zeng et al. (2016), the New York Stock Exchange is non-fractal, which means it is vulnerable to the absence of important stocks. Moradi et al. (2021) investigated the validity of the fractal market hypothesis in the Tehran and London Stock Exchanges. They showed that fractal market hypotheses are acceptable for both stock exchanges. Using the MF-DFA model, Xu et al. (2021) analyzed the multifractal properties of ten sectors on the Shanghai Stock Exchange. They reported that all sectors had low market efficiency and higher multifractality during the COVID-19. Arashi and Rounag (2022) investigated the NASDAQ stock exchange’s multifractal property and discovered that it has a nonlinear and chaotic structure.

Some studies have investigated market efficiency on a sectoral level. For instance, Cheong (2008) examined a structural break unit root test to investigate nine Malaysian sectoral indices’ weak form market efficiency. They found that all sectoral indices are not inefficient in the face of structural change. Tiwari et al. (2017) used the MF-DFA method to determine the multifractal features of Dow Jones sector Exchange-Traded Fund (ETF) indices. They exhibited that sector ETF indexes are multifractal in nature, with consumer goods and utilities being more efficient than telecommunications and finance. They also expressed that market efficiency has decreased since the Global Financial Crisis. Stosic et al. (2019) examined BOVESPA and seven sectors including Consumer Stock Index, Electric Utilities Index, Financials Index, Basic Materials Index, Real Estate Index, Industrials Index, and Public Utilities Index by using the MF-DFA model. They showed that different multifractal features exist for different sectors due to their unique dynamics. Furthermore, they found that the public and electric utility sectors are less efficient, whereas other sectors are more efficient. Arshad et al. (2020) examined the effect of Brexit on the volatility and efficiency of the London Stock Exchange as well as six sectoral indices. They concluded that market efficiency decreased during the Brexit vote period. Diallo et al. (2021) investigated the market efficiency of seven sectoral indices in the West African Economic and Monetary Union. They found that all sectoral indices have multifractal features and that, except for the agriculture sector, the null hypothesis of a weak form of an efficient market is rejected. Çatık et al. (2020) examined the time-varying effect of oil prices on Turkish sectoral stock returns. They stated that the alleged effect varies significantly over time. Furthermore, they detected that oil prices harm banking, transportation, chemicals, food and beverage, electricity, metal goods, industrials, and machinery. Fernandes et al. (2021) researched the effect of COVID-19 on 26 Chinese sectoral indices and find that COVID-19 increases inefficiency in the majority of these sectors, with the effects varying depending on the economic sector. Caporale et al. (2021) investigated the effect of US policy responses on sectoral indices in the United States during the COVID-19 period. The results provide evidence of mean reversion in seven sectoral stock indices: consumer staples, consumer discretionary, industrial, technology, health, telecommunications, and utilities. However, the energy, basic materials and real estate indices are highly persistent. Besides, they indicated that the federal fund rate, and monetary and fiscal announcements affect positively all sectors except industrial and energy, whereas COVID-19 negatively impacts most of these indices. Akdeniz et al. (2021) investigated the impact of COVID-19 on oil–gas sectoral indices in France, Italy, Spain, China, the United Kingdom, and the United States. They reported that the oil–gas sector’s market risk affects all countries during the COVID-19 period, and the oil price factor negatively impacts all countries except China. Caporale et al. (2022) explored the effects of exchange rates and oil prices on BRICS-T sectoral indices. They determined that the price of oil positively affects the energy sector in all countries except India, but a negative effect on the financial and transportation sectors in BRIS countries, Turkey, and India. Oil prices harm the industrial sectors of all countries except Turkey.

## Data

**Fig. 1:**
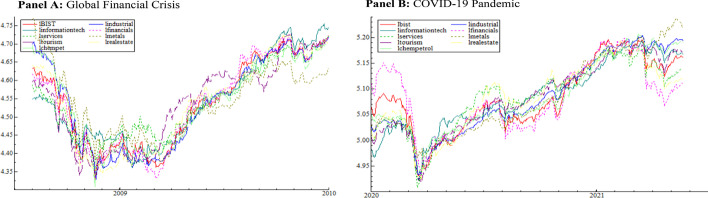
Logarithmic close prices matching series by mean over the periods of the GFC and Covid-19 outbreak

**Fig. 2:**
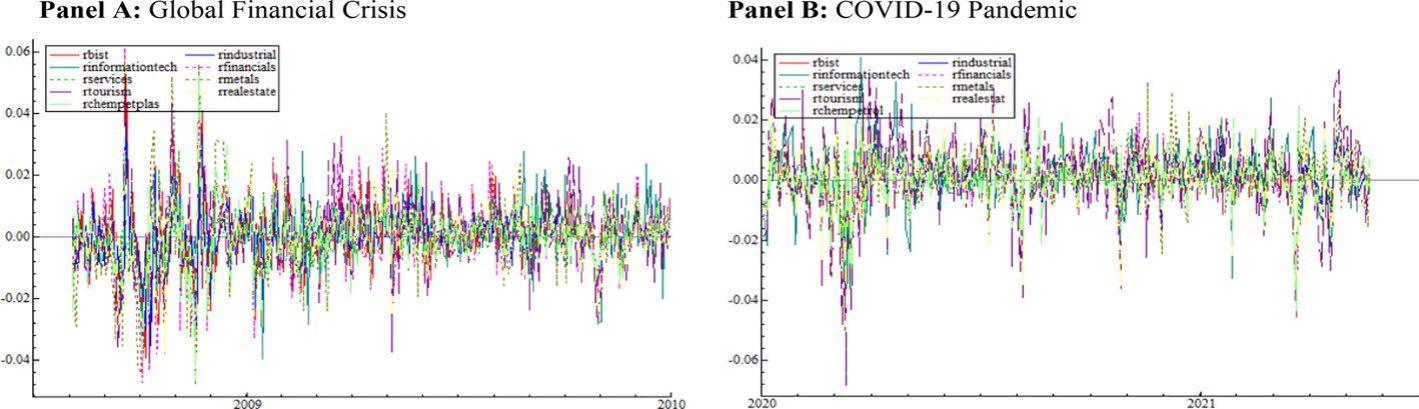
Return series over the periods of the GFC and COVID-19 outbreak

We consider daily data, including the Borsa Istanbul (XU) 100 (BIST 100) index and the eight sector indices, which are industrials, financials, services, information technology, basic metals, tourism, real estate investment, and chemical, petroleum, and plastic. Stock return multifractality is frequently used as a measure of market inefficiency (Cajueiro et al. 2009; Zhou 2009). These characteristics may differ in sectoral returns (Mensi et al. 2017). In other words, because of their unique structures, sectoral returns have varying levels of market efficiency. Furthermore, sectoral returns provide important information about the behavior of individual industries (Choi 2021). As a result, the study takes into account sectoral returns. These sectors are preferred because their gross domestic product (GDP) weights are quite high and they are highly sensitive to international developments. In 2021, the shares of GDP invested in industrials, financials, services, information technology, tourism, and real estate are 21.8%, 3.1%, 24.4%, 2.6%, 4.6%, and 6.5%, respectively. Due to information inefficiency and capital flows from the developed market, investors in emerging markets can earn higher profits than those in developed markets. Turkey is one of the largest economies in Eastern Europe and the Middle East among emerging markets (Vardar et al. 2012). In addition, with a trading volume of 794 billion dollars, Borsa Istanbul is ranked 20th among global stock exchanges in 2021. It is one of the most liquid markets in the world, with a turnover rate of 5%. Borsa Istanbul represents emerging markets well due to its so-called characteristics. The Borsa Istanbul Stock Exchange’s main index is the BIST 100. It consists of 100 stocks chosen from among those traded on the Stars Market. In other words, the BIST 100 index is a fundamental indicator used to assess the current state of 100 stocks with the highest market and trading volumes. The data are obtained from the investing.com website. The sample data span two time periods: the GFC and the COVID-19 outbreak. The first comprises the years 23.08.2007–16.09.2009, whereas the second spans the years 02.01.2020–28.01.2022. The formula for calculating the return series is as follows:

1 $$r_{t} = \ln \left( {P_{t} /P_{t - 1} } \right)$$

where $P_{t}$ is the close price for the relating stock market index. Figure 1 shows the daily logarithmic BIST 100 index and sector indices matched by mean for the COVID-19 outbreak and the GFC. Figure 2 depicts the return series calculated by Eq. (1) during the COVID-19 outbreak and the Great Recession. As illustrated in Fig. 1, close prices for all sectors fell sharply during both periods. Similarly, Fig. 2 shows that the return series deviated significantly from their averages during both periods.

Table 1 reveals the descriptive statistics for the return series, which include the BIST 100 index and selected sectoral indices for the GFC and COVID-19 outbreak periods. According to Table 1, the services and tourism sector indices had the highest average returns during the GFC and COVID-19 outbreaks, respectively. According to the standard deviation values, the basic metals sector and the tourism sector have the highest risk during the periods of the GFC and the COVID-19 outbreak, respectively. The skewness and kurtosis values for all sectors are greater than the critical values for the normal distribution, which are “0” and “3,” respectively. Similarly, the null hypothesis assuming that the returns distribute normally for the Jarque–Bera test is rejected at level 5%. Therefore, the BIST 100 index and sectoral indices have a leptokurtic distribution. RBIST, RFINANCIALS, RSERVICES, and RMETALS had positive skewness values during the GFC period, indicating that their distributions were right-skewed. Meanwhile, the others have negative skewness values, indicating a left-skewed distribution. However, due to negative skewness values during the COVID-19 outbreak period, all variables have a left-skewed distribution. This result suggests that extreme events occurred for all indices during the COVID-19 period.

**Table 1:** Descriptive statistics relevant to the aggregate and sectoral stock returns during the GFC and the COVID-19 Outbreak

	Mean	Median	Maximum	Minimum	Std. Dev	Skewness	Kurtosis	Jarque–Bera	N
*Panel A: the GFC*									
RBIST	0.00003	− 0.00012	0.05267	− 0.03915	0.01010	0.08534	5.50460	136.80***	521
RINDUSTRIAL	− 0.00004	0.000011	0.03643	− 0.03516	0.00810	− 0.36795	5.67120	166.65***	521
RFINANCIALS	0.00004	− 0.00004	0.06133	− 0.04725	0.01208	0.08439	5.34716	120.21***	521
RSERVICES	0.00013	0.00014	0.04340	− 0.03090	0.00832	0.18078	5.46723	134.98***	521
*RINFORMATION*									
TECH	− 0.00003	0.00032	0.04910	− 0.03971	0.00915	− 0.25259	6.64768	294.38***	521
RMETALS	− 0.00003	− 0.00013	0.05712	− 0.04806	0.01320	0.15520	5.32365	119.30***	521
RTOURISM	− 0.00035	− 0.00057	0.04644	− 0.04689	0.01129	− 0.01138	5.59722	146.44***	521
RREALESTATE	− 0.00017	0.00020	0.03670	− 0.03709	0.00859	− 0.48033	5.28166	133.04***	521
RCHEMPETPLAS	− 0.00001	0.00003	0.05231	− 0.04666	0.00971	− 0.31495	6.46511	269.26***	521
*Panel B: the COVID-19 outbreak*									
RBIST	0.00028	0.00081	0.02523	− 0.04475	0.00723	− 1.55604	10.5141	956.37***	521
RINDUSTRIAL	0.00080	0.00133	0.02725	− 0.04411	0.00745	− 1.65105	10.3738	943.79***	521
RFINANCIALS	0.000001	0.00024	0.03029	− 0.04478	0.00857	− 0.93327	7.43105	334.25***	521
RSERVICES	0.00032	0.00078	0.01979	− 0.04371	0.00687	− 1.74026	11.3833	1191.2***	521
*RINFORMATION*									
TECH	0.00117	0.00137	0.04074	− 0.06671	0.01155	− 0.98469	7.78335	386.88***	521
RMETALS	0.00097	0.00102	0.03240	− 0.04557	0.00885	− 0.39026	6.05293	143.56***	521
RTOURISM	0.00138	0.00251	0.03667	− 0.06835	0.01375	− 0.82702	5.59460	136.88***	521
RREALESTAT	0.000445	0.000892	0.031351	− 0.0513	0.009723	− 1.12728	7.32664	344.15***	521
RCHEMPETROL	0.000711	0.000954	0.028453	− 0.04316	0.008132	− 0.9465	7.25489	313.56***	521

***Shows a significant level at 1%

## Methodology

### Multifractal detrend fluctuation analysis (MF-DFA) procedure

A multifractal structure can be found in many financial time series, such as stock market returns (Mandelbrot 1999; Bouchaud et al. 2000; Kwapien et al. 2005). Multifractal models also consider significant aspects of price patterns unlike other traditional models (Lux 2003; Eisler and Kertesz 2004). Furthermore, essential observables in emerging market dynamics exhibit richer multifractality (Jin and Lu 2006; Eisler and Kerteszi 2007). Therefore, it is important to test the stage of market development using multifractal measures. This is because information about market inefficiency is important to policymakers, risk managers, and investors. Because an inefficient market causes resource allocation and economic distortions.

In this study, the MF-DFA is used to investigate the multifractality (or long memory) properties in non-stationary financial time series and to determine the financial market efficiency (Kantelhardt et al. 2002; Mensi et al. 2017; Choi 2021). The level of persistency, long memory, and random walk behaviors in financial asset prices can be analyzed using this method (Mensi et al. 2017).

The MF-DFA method consists of five steps (Kantelhardt et al. 2002). Assume that $x_{k}$ is a stock market price with length N.

*Step 1* Define the “profile”

2 $$Y\left( i \right) = \mathop \sum \limits_{k = 1}^{i} \left[ {x_{k} - \overline{x}} \right], i = 1, \ldots , N$$

*Step 2* Divide the profile $Y\left( i \right)$ into the non-overlapping segments (${\text{N}}_{{\text{s}}} \equiv {\text{int}}\left( {{\text{N}}/{\text{s}}} \right)$, where s equals to length). Because the length N is frequently not a multiple of the time scale s, the procedure is repeated from the beginning of the sample to the end. Therefore, 2 $N_{s}$ segments are obtained.

*Step 3* For each of the 2 $N_{s}$ segments, it is calculated the local trend. Specify the variance for each segment $v, v = 1, \ldots , N$

3 $$F^{2} \left( {v,s} \right) = \frac{1}{s}\mathop \sum \limits_{i = 1}^{s} \left\{ {Y\left[ {\left( {v - 1} \right)s + i} \right] - y_{v} \left( i \right)} \right\}^{2}$$

For $$v = N_{s} + 1, \ldots , 2N_{s}$$

4 $$F^{2} \left( {v,s} \right) = \frac{1}{s}\mathop \sum \limits_{i = 1}^{s} \left\{ {Y\left[ {N - \left( {v - N_{s} } \right)s + i} \right] - y_{v} \left( i \right)} \right\}^{2}$$

where $y_{v} \left( i \right)$ is the fitting polynomial in segment v. Linear, quadratic, cubic, or higher order polynımials can be used in the fitting procedure.

*Step 4* Calculate the qth order fluctuation function by averaging over all segments

5 $$F_{q} \left( s \right) = \left\{ {\frac{1}{{2N_{s} }}\mathop \sum \limits_{v = 1}^{{2N_{s} }} \left[ {F^{2} \left( {v,s} \right)} \right]^{q/2} } \right\}^{1/q}$$

where q is the index variable and can take any real value. In the case of $q = 2$, the standard DFA process is retrieved. If q is equal to zero ($q = 0$), it is gone to the fifth step. $F_{q} \left( s \right)$ depends on s for different q. Thus, steps 2–4 must be repeated for several s. $F_{q} \left( s \right)$ will rise with increasing s.

*Step 5* Detect the scaling behavior of the fluctuation functions by analyzing the log–log plots $F_{q} \left( s \right)$ for each value of q.

If series $x_{i}$ correlate with long-range $F_{q} \left( s \right)$ rises for large values of s, as a power law:

6 $$F_{q} \left( s \right)\sim s^{h\left( q \right)}$$

where the $h\left( q \right)$ is based on q. For the stationary time series, h (2) is known as the Hurst exponent. Therefore, $h\left( q \right)$ is referred as the generalized Hurst exponent. If $h\left( 2 \right) = 0.5$, it states that the series does not correlate and have a random walk. If $0.5 < h\left( 2 \right) < 1$, it represents the long memory. However; If $0 < h\left( 2 \right) < 0.5$, it tends to the mean-reverting process.

### Overlap moving window

Dividing the profile into non-overlapping segments can result in new pseudo-fluctuation errors due to discontinuities in the polynomial fit at the split data junction points, resulting in distortions in the scale exponent estimates. We use the overlap moving window algorithm to overcome the problem of data segmentation discontinuity and provide better statistics for shorter time scales. First, the moving window size for the time series with N observations is determined. Second, the step size for each forward movement is determined. Therefore, the number of movement times is calculated by subtracting the moving window size from the total length of the time series data (Zhang et al. 2019; Gorjão et al. 2022).

## Empirical findings

We use the MF-DFA to calculate aggregate and sectoral market efficiency based on the degree of multifractality and the size of the multifractal spectrum. A higher level of multifractality indicates stronger multifractality, implying a decline in market efficiency in the relevant sector.

First, we use the Lee-Strazcich unit root test with two structural breaks to determine whether the so-called variables are stationary. Table 2 displays the results. The null hypothesis of non-stationarity is rejected for all return series, according to Table 2. The breaking dates correspond to the bankruptcy of Lehman Brothers and the appearance of the first COVID-19 cases. The series’ nonlinear structure is then tested. As a result, for the so-called return series, we employ the Teraesvirta, White, Keenan, Tsay, and likelihood ratio tests. Table 3 summarizes the results. According to Table 3, the null hypothesis of linearity is rejected for all series. In other words, their structure is nonlinear.

**Table 2:** Lee–Strazicich unit root test

Model A (Crash)					Critical values	Model C (Constant and trend)						Critical values
	LM	Lag	Breaking dates			LM	Lag	Breaking dates				
			D1t	D2t	%5			D1t	DT1t	D2t	DT2t	%5
*GFC period*												
RBIST	− 4.7103	5	4.11.2008	11.9.2009	− 3.5630	− 6.8514	5	4.12.2008	4.12.2008	26.1.2009	26.1.2009	− 6.1080
RINDUSTRIAL	− 5.4965	0	23.9.2008	13.1.2009	− 3.5630	− 6.2979	0	4.12.208	4.12.2008	23.1.2009	23.1.2009	− 6.1080
RINFORMATION	− 5.7276	0	9.10.2008	6.5.2009	− 3.5630	− 6.5685	0	23.2.2009	23.2.2009	14.4.2009	14.4.2009	− 6.2680
RFINANCIALS	− 4.7026	1	16.10.2008	7.1.2009	− 3.5630	− 6.6244	1	26.9.2008	26.9.2008	29.12.2008	29.12.2008	− 6.1080
RSERVICES	− 6.5011	0	16.10.2008	4.2.2009	− 3.5630	− 6.3119	0	29.9.2008	29.9.2008	26.11.2008	26.11.2008	− 6.1080
RMETALS	− 5.5332	0	26.9.2008	24.4.2009	− 3.5630	− 6.3218	0	25.9.2008	25.9.2008	19.2.2009	19.2.2009	− 6.3120
RTOURISM	− 4.6253	0	9.10.2008	3.6.2009	− 3.5630	− 6.7539	0	23.9.2008	23.9.2008	13.11.2008	13.11.2008	− 6.1080
RREALESTATE	− 4.7970	0	7.1.2009	6.5.2008	− 3.5630	− 6.8308	0	26.9.2008	26.9.2008	20.11.2008	20.11.2008	− 6.1080
RCHEMPETROL	− 5.2036	1	7.10.2008	4.12.2008	− 3.5630	− 6.5727	1	18.11.2008	18.11.2008	23.1.2009	23.1.2009	− 6.1080
*COVID-19 period*												
RBIST	− 5.6192	1	7.1.2020	21.5.2021	− 3.5630	− 10.2027	1	12.3.2020	12.3.2020	11.8.2020	11.8.2020	− 6.3120
RINDUSTRIAL	− 5.7433	1	16.3.2020	22.3.2021	− 3.5630	− 9.9880	1	17.3.2020	17.3.2020	6.8.2020	6.8.2020	− 6.3120
RINFORMATION	− 4.0343	1	19.3.2020	22.3.2021	− 3.5630	− 6.9514	1	11.3.2020	11.3.2020	30.3.2021	30.3.2021	− 5.9170
RFINANCIALS	− 6.0347	1	18.3.2020	6.8.2020	− 3.5630	− 10.4502	1	27.2.2020	27.2.2020	13.8.2020	13.8.2020	− 6.3120
RSERVICES	− 5.4437	1	23.3.2020	22.3.2020	− 3.5630	− 9.4254	1	6.3.2020	6.3.2020	2.11.2020	2.11.2020	− 6.1850
RMETALS	− 6.8597	1	17.3.2020	17.11.2020	− 3.5630	− 10.4034	1	11.3.2020	11.3.2020	10.8.2020	10.8.2020	− 6.3120
RTOURISM	− 4.5718	1	16.3.2020	6.8.2020	− 3.5630	− 8.9689	1	11.3.2020	11.3.2020	22.3.2021	22.3.2021	− 5.9170
RREALESTATE	− 5.6951	1	16.3.2020	6.8.2020	− 3.5630	− 10.8210	1	16.3.2020	16.3.2020	22.3.2021	22.3.2021	− 5.9170
RCHEMPETROL	− 5.4966	1	16.3.2020	22.3.2021	− 3.5630	− 8.8731	1	17.3.2020	17.3.2020	28.7.2020	28.7.2020	− 6.3120

Critical values are obtained from Lee and Strazicich (2003)

**Table 3:** Nonlinearity test

	Teraesvirta	White	Keenan	Tsay	LR
RBIST	1.5536 (0.4598)	0.0266 (0.9867)	3.3048 (0.0691)	4.2601 (0.0051)	3.1302 (0.0511)
RINDUSTRIAL	1403.008 (0.0000)	1155.03 (0.0000)	616.8418 (0.0000)	7.8849 (0.0000)	614.558 (0.0000)
RINFORMATION	1396.843 (0.0000)	841.7639 (0.0000)	828.2828 (0.0000)	18.5397 (0.0000)	804.549 (0.0000)
RFINANCIALS	1370.638 (0.0000)	956.8247 (0.0000)	729.2311 (0.0000)	11.0627 (0.0000)	707.8793 (0.0000)
RSERVICES	1405.297 (0.0000)	1284.147 (0.0000)	549.5759 (0.0000)	5.2320 (0.0000)	559.1786 (0.0000)
RMETALS	1373.058 (0.0000)	1342.792 (0.0000)	761.5324 (0.0000)	13.9666 (0.0000)	736.4937 (0.0000)
RTOURISM	1389.588 (0.0000)	583.2149 (0.0000)	878.0924 (0.0000)	24.8813 (0.0000)	889.8795 (0.0000)
RREALESTATE	1425.581 (0.0000)	1221.735 (0.0000)	747.1507 (0.0000)	13.6214 (0.0000)	735.3947 (0.0000)
RCHEMPETROL	1402.947 (0.0000)	1300.331 (0.0000)	686.7822 (0.0000)	8.9187 (0.0000)	696.1988 (0.0000)

The values in the parenthesis indicate the probabilities

Table 4 shows the results of the long-memory and asymmetric unit root tests. According to long memory tests conducted by Geweke and Porter-Hudack (1983) and Robinson and Henry (1999), d values for all variables for both the GFC and COVID-19 periods are between 0 and 0.5. This result shows that all returns have long memory. When the statistics of the KSS (2003) nonlinear unit root test, which is based on the ESTAR process, are examined, we determined that all return series are globally stationary ESTAR processes at the 5% level.

**Table 4:** The results of long memory and nonlinear unit root tests

	Geweke and Porter-Hudack (1983) test		Robinson and Henry (1999) test		KSS unit root test	
	d	*p*-value	d	*p*-value	Statistic	Critical value (%5)
*Panel A: the GFC*						
RBIST	0.1008	0.0751	0.0938	0.0125	− 3.2881	− 2.93
RINDUSTRIAL	0.1252	0.0270	0.1460	0.0375	− 4.5641	− 2.93
RFINANCIALS	0.1123	0.0473	0.0815	0.0300	− 3.3959	− 2.93
RSERVICES	0.0169	0.0643	0.0285	0.0480	− 2.9840	− 2.93
RINFORMATION TECH	0.1142	0.0436	0.1094	0.0036	− 3.4788	− 2.93
RMETALS	0.1516	0.0074	0.1460	0.0001	− 3.9961	− 2.93
RTOURISM	0.1303	0.0214	0.1054	0.0050	− 4.7485	− 2.93
RREALESTATE	0.1248	0.0274	0.1537	0.0000	− 2.9412	− 2.93
RCHEMPETPLAS	0.1394	0.0138	0.1271	0.0007	− 3.1307	− 2.93
*The COVID* − *19 outbreak*						
RBIST	0.0679	0.2356	0.0472	0.2142	− 3.1661	− 2.93
RINDUSTRIAL	0.1368	0.0169	0.0667	0.0792	− 3.9240	− 2.93
RFINANCIALS	0.0619	0.0796	0.0500	0.0881	− 3.2987	− 2.93
RSERVICES	0.0978	0.0878	0.0586	0.0829	− 4.4558	− 2.93
RINFORMATIONTECH	0.1781	0.0019	0.1746	0.0000	− 5.4153	− 2.93
RMETALS	0.0421	0.0623	0.0116	0.0600	− 3.9878	− 2.93
RTOURISM	0.2193	0.0001	0.1439	0.0002	− 4.8779	− 2.93
RREALESTATE	0.1272	0.0264	0.0780	0.0401	− 6.3491	− 2.93
RCHEMPETPLAS	0.1657	0.0038	0.0776	0.0411	− 3.2916	− 2.93

**Fig. 3:**
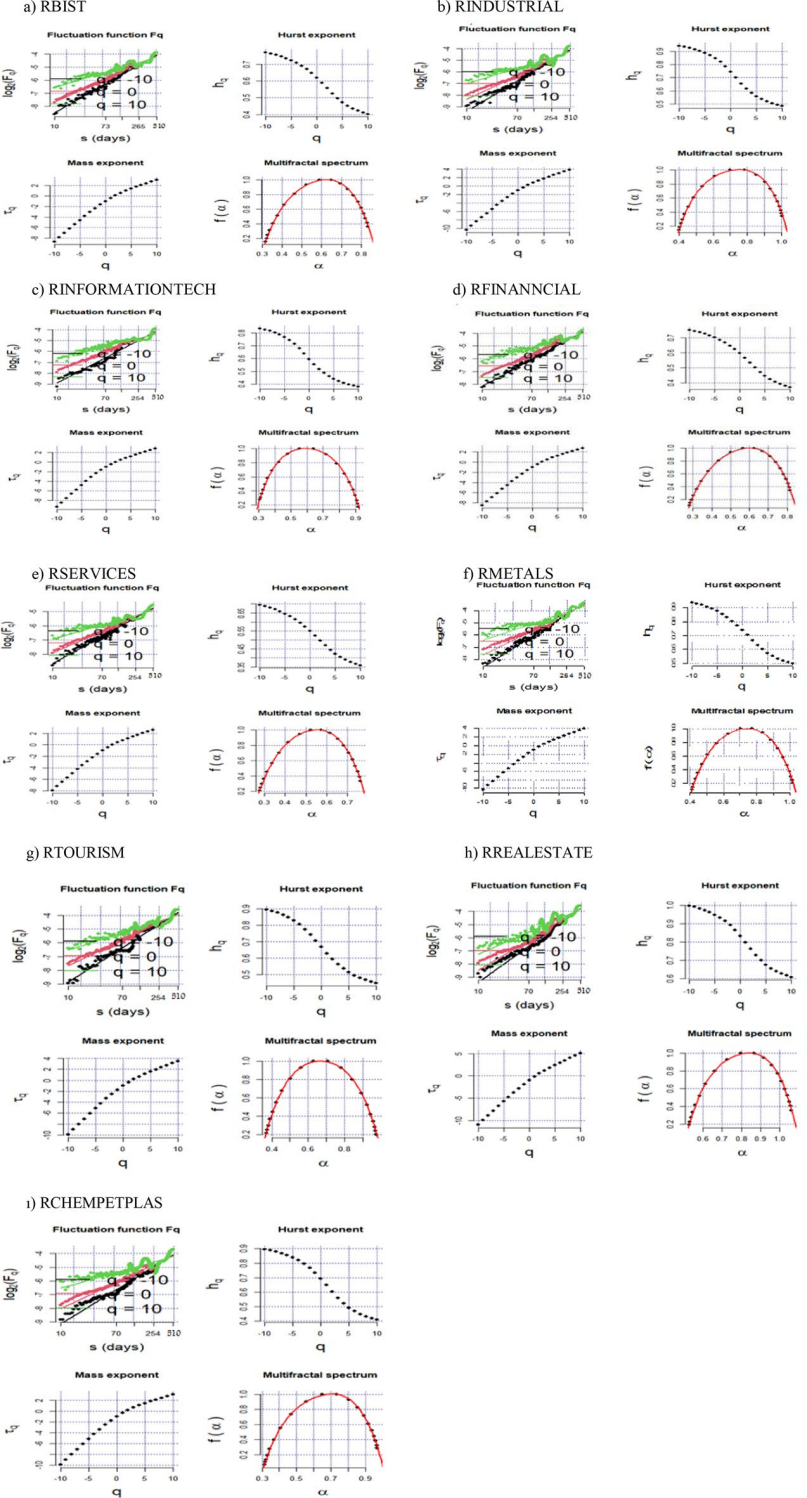
The curves of multifractal fluctuation functions Fq(s) to s in the log–log plot, Generalized Hurst exponents h(q), Mass exponent and multifractal spectra for the aggregate and sectoral indices during the GFC period. Note: Fq(s) indicates variation in the fluctuation of series for various orders across various time segments. h(q) indicates the scaling exponent which is the slope of the regression for log(Fq(s)) to s. τ(q), calculated as qh(q)-1, is multifractal exponents which have a nonlinear structure if the series follows a multifractal structure. f(α), called as singularity spectrum, indicates the fractality dimension of the subperiod related to the series

**Fig. 4:**
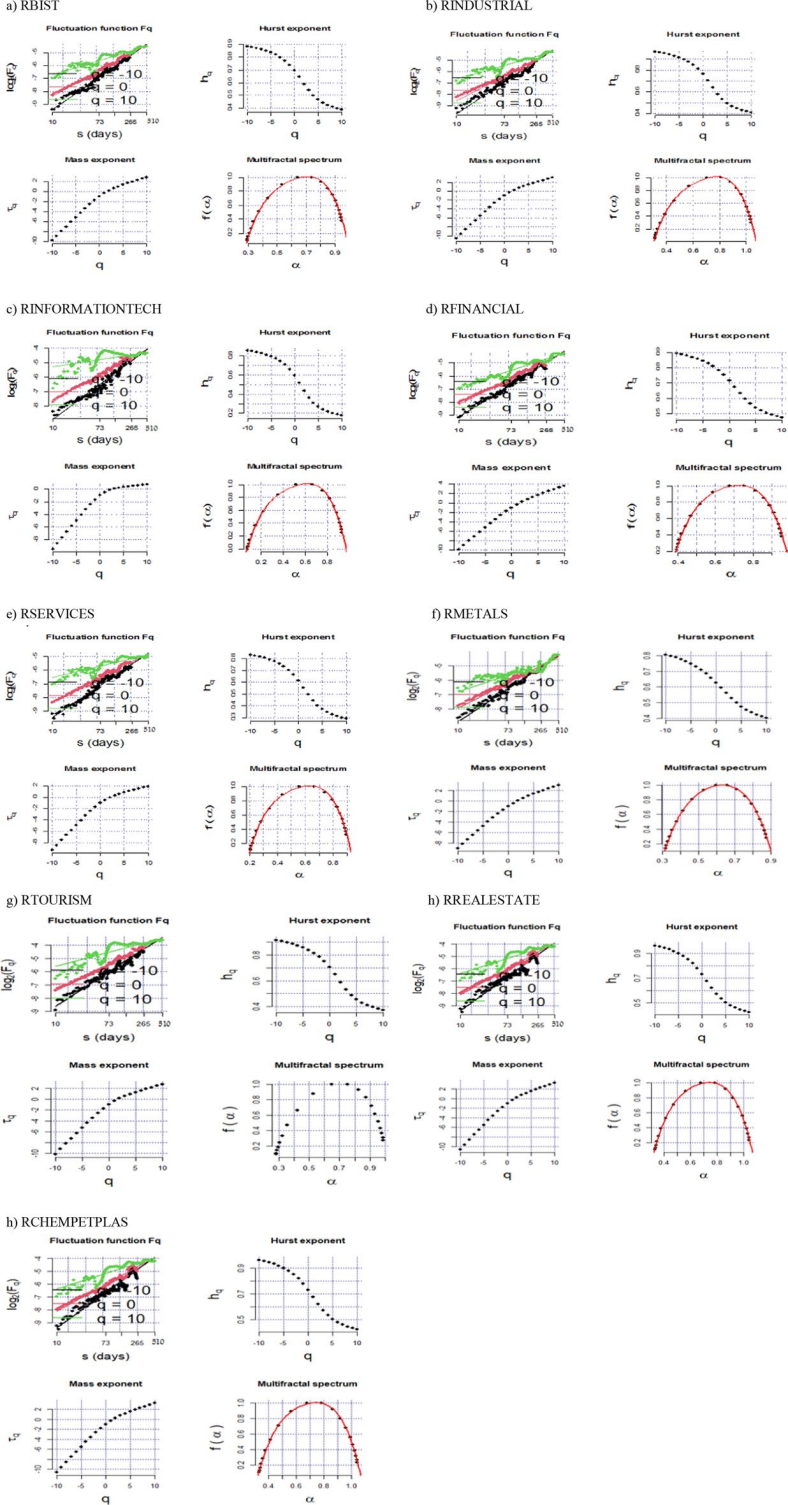
The curves of multifractal fluctuation functions Fq(s) to s in the log–log plot, Generalized Hurst exponents h(q), Mass exponent and multifractal spectra for the aggregate and sectoral indices during the COVID-19 outbreak. Note: Fq(s) indicates variation in the fluctuation of series for various orders across various time segments. h(q) indicates the scaling exponent which is the slope of the regression for log(Fq(s)) to s. τ(q), calculated as qh(q)-1, is multifractal exponents which have a nonlinear structure if the series follows a multifractal structure. f(α), called as singularity spectrum, indicates the fractality dimension of the subperiod related to the series

Figures 3 and 4 show the log–log plot curves of the multifractal fluctuation functions Fq(s) to s, the generalized Hurst exponents h(q), the mass exponent, and the multifractal spectra for the aggregate and sectoral indices during the GFC and COVID-19 outbreak, respectively. The log–log plot between the long scale and the order of fluctuation function represents the fractality of the aggregate and sectoral stock returns. The scaling range is important in detecting a linear structure because of restricting the lower and upper levels. As shown in Figs. 3 and 4, $log_{2} F\left( q \right)$ increases for high values, implying the existence of scaling laws and exponents for aggregate and sectoral returns during the two crisis periods. The h(q) plots show a decremental trend, supporting the so-called returns’ multifractality. Likewise, the nonlinear relationship between τ(q) and q validates multifractality for all returns. A multifractality of a financial series can be analyzed using the singularity spectrum f(α). f(α) shows the fractal dimension of the subset of the financial series, whereas α indicates the singularity strength. A financial series with a narrower multifractal spectrum is more efficient, with less heterogeneity and market risk (Zunino et al. 2008). The right side of the spectrum between f(α) and α demonstrates the small fluctuations, whereas the left side reflects the large fluctuations. A multifractal is a multifractal spectrum of a financial series’ power law exponent. This means that the width and shape of a spectrum can alter financial series with different scales (Zhu and Zhang 2018). The small fluctuations for all returns are persistent and have a positive long memory, as seen in the spectra for the GFC and the COVID-19 outbreak periods. However; the large fluctuations tend to mean-revert and have an anti-persistent structure in the long run. During the GFC, the spectrum for RBIST, RINFORMATIONTECH, RFINANCIALS, and RSERVICES is centered at 0.6, whereas the spectrum for RTOURISM and RCHEMPETPLAS is between 0.6 and 0.7. However, RINDUSTRIAL, RMETALS and RREALESTATE have the highest spectrum, which is between 0.7 and 0.8 in the so-called period. The highest spectra means that RINDUSTRIAL, RMETALS, and RREALESTATE exhibit higher multifractal behavior and market risk. Meanwhile, RINFORMATIONTECH, RSERVICES, and RMETALS have a spectrum centered at 0.6 in the COVID-19 period, but RTOURISM has a spectrum equal to 0.7. During the same time period, RBIST, RINDUSTRIAL, RFINANCIALS, RREALESTATE, and RCHEMPETPLAS have a spectrum between 0.7 and 0.8, indicating that these sectors have greater multifractal behavior.

For $q \le 0$, the h(q) values for all returns exceed 0.5 during the GFC and the COVID-19 outbreak. This result implies that, despite the small fluctuations during the two crisis periods, all sectoral returns have long-term persistence. As considered $q \le 2$, the values of h(q) are higher than 0.5, except for the services, metals, and information technology sectors during the GFC, and the information technology sector during the COVID-19 outbreak. Furthermore, due to the characteristics of each crisis, the persistence levels of sectoral returns change for the two crisis periods. In other words, the GFC has resulted from financial issues, whereas an outbreak caused the recent crisis. Throughout the COVID-19 outbreak, the persistence for all sectors is greater than during the GFC.

Tables 5 and 6 demonstrate the h(q) slopes for scales ranging from − 5 to 5 for the GFC and COVID-19 outbreaks, respectively. If $0 \le h\left( q \right) \le 0.5$, the series exhibits fractal behavior and market efficiency cannot be obtained. In such a case, the series tends to mean-revert, making the market riskier for investors. The return has a negative or anti-persistence long memory, which means that the movements in the so-called series differ significantly from those observed in a random series. If $h\left( q \right) = 0.5$, the series is random, implying that the market is efficient. This indicates that returns do not have a long memory and that investors cannot defeat the market. If $0.5 < h\left( q \right) \le 1$, the series has a fractal and persistent structure, which is incompatible with market efficiency, and the returns have a long memory (Patil and Rastogi 2020). As shown in Tables 4 and 5, the h(q) values vary with q for all sectoral indices, indicating that all return series exhibit multifractal behavior. In addition, h(q) for small fluctuations is higher than h(q) for large fluctuations in all sectoral indices, indicating that the dynamics of the interested markets are more persistent and exhibit a long-memory characteristic more clearly in small fluctuations. For q =  − 5, the real-estate sector has the highest persistence, whereas the services sector has the lowest persistence during the GFC. During the COVID-19 outbreak, the industrial sector has the strongest persistence for q =  − 5, whereas the metals sector has the weakest. These results suggest that for the smallest fluctuations, the real-estate sector during the GFC and the industrial sector during the COVID-19 outbreak exhibit long-range dependence, which explains the multifractality of the real-estate sector during the GFC period and the industrial sector during the COVID-19 outbreak on a large scale.

**Table 5:** Generalized Hurst exponents relevant to the aggregate and sectoral returns for q varying from − 5 to 5 for the GFC

q	RBIST	RINDUSTRIAL	RINFORMATION TECH	RFINANCIAL	RSERVICES	RMETALS	RTOURISM	RREAL ESTATE	RCHEMPETPLAS
− 5	0.7228	0.8878	0.7674	0.7007	0.6457	0.8749	0.8279	0.9494	0.8392
− 4	0.7079	0.8696	0.7435	0.685	0.6309	0.8541	0.8041	0.9336	0.8198
− 3	0.6904	0.8472	0.7141	0.667	0.614	0.8299	0.7753	0.9148	0.7962
− 2	0.6701	0.8194	0.6788	0.6463	0.5949	0.8026	0.7418	0.8924	0.7674
− 1	0.6466	0.7853	0.6486	0.6727	0.5736	0.7724	0.7051	0.8656	0.7328
0	0.6194	0.7447	0.5961	0.596	0.5499	0.7392	0.6672	0.8342	0.6924
1	0.5889	0.6999	0.5948	0.5661	0.524	0.7135	0.6299	0.7991	0.6474
2	0.5565	0.6554	0.5778	0.5838	0.4969	0.6665	0.5948	0.763	0.6011
3	0.5248	0.616	0.4863	0.5513	0.4699	0.6342	0.5634	0.7293	0.5577
4	0.4964	0.5834	0.4605	0.4714	0.445	0.5999	0.5365	0.7003	0.5204
5	0.4722	0.5573	0.4395	0.4457	0.4233	0.5738	0.5141	0.6763	0.4901

**Table 6:** Generalized Hurst exponents relevant to the aggregate and sectoral returns for q varying from − 5 to 5 for the COVID-19 outbreak

q	RBIST	RINDUSTRIAL	RINFORMATION TECH	RFINANCIAL	RSERVICES	RMETALS	RTOURISM	RREAL ESTATE	RCHEMPETPLAS
− 5	0.8775	0.8927	0.8789	0.867	0.8453	0.7336	0.8553	0.8945	0.8283
− 4	0.8613	0.8743	0.8658	0.8516	0.8271	0.7128	0.8456	0.8768	0.8143
− 3	0.843	0.8541	0.8525	0.8335	0.8065	0.6878	0.8366	0.8554	0.7991
− 2	0.8225	0.8334	0.84	0.8125	0.7843	0.6585	0.8297	0.8297	0.7834
− 1	0.8001	0.8138	0.8386	0.7885	0.7618	0.6251	0.826	0.7986	0.7673
0	0.7752	0.7937	0.8163	0.7621	0.7388	0.5884	0.822	0.759	0.7489
1	0.747	0.7675	0.7991	0.7346	0.7119	0.5504	0.8079	0.7058	0.7034
2	0.7164	0.7329	0.775	0.7076	0.6795	0.5135	0.7793	0.6418	0.6897
3	0.6861	0.696	0.7471	0.6825	0.6455	0.4801	0.7447	0.5822	0.6531
4	0.659	0.6628	0.7202	0.6604	0.6145	0.4514	0.7129	0.5353	0.6195
5	0.6361	0.6351	0.6967	0.6415	0.5883	0.4274	0.6865	0.5002	0.5914

To compare the aggregate and sectoral efficiency during the GFC and the COVID-19 outbreak, we calculate the market deficiency measure (MDM) as follows (Wang et al. 2009):

7 $$MDM = \frac{1}{2}\left( {\left| {h\left( { - 5} \right) - 0.5} \right| + \left| {h\left( 5 \right) - 0.5} \right|} \right) = \frac{1}{2}\Delta h$$

If the financial market is efficient, all small and large fluctuations follow a random walk process. In an efficient market, the MDM values will be equal to zero. In an inefficient market, however, the so-called value is higher. Tables 7 and 8 show the MF-DFA rankings for different q and the average of MF-DFA rankings for the aggregate and sectoral stock indices, which demonstrates a market’s ranking efficiency. The services sector during the GFC and the metals sector during the COVID-19 outbreak have the lowest MDM values (0.074 and 0.1122, respectively), implying that these are the most efficient markets. However, during the GFC period, the real estate sector had the highest MDM values (0.3223), followed by the metals and industrial sectors, whereas during the COVID-19 period, the information technology sector had the highest MDM values (0.3013), followed by the tourism and industrial sectors. Therefore, these markets can be described as the most inefficient. This disparity in market efficiency levels results from variations in the persistent improving level and can be considered by investors when managing portfolio risk and allocating assets. These results clearly show that real estate bubbles caused the GFC; however, with the COVID-19 outbreak, an extreme increase in the information technology market was observed, indicating the presence of a bubble in the so-called sector.

**Table 7:** The MF-DFA rankings of the aggregate and sectoral market returns, which indicates the measure of market efficiency using MDM

The GFC			The COVID-19 outbreak		
Ranking	Sector	MDM	Ranking	Sector	MDM
*h(q): − 5,5*					
1	RREALESTATE	0.3128	1	RINFORMATIONTECH	0.2878
2	RMETALS	0.2243	2	RTOURISM	0.2709
3	RINDUSTRIAL	0.2225	3	RINDUSTRIAL	0.2639
4	RCHEMPETPLAS	0.1745	4	RBIST	0.2568
5	RTOURISM	0.1710	5	RFINANCIAL	0.2542
6	RINFORMATIONTECH	0.1639	6	RSERVICES	0.2168
7	RFINANCIAL	0.1275	7	RCHEMPETPLAS	0.2098
8	RBIST	0.1253	8	RREALESTATE	0.1973
9	RSERVICES	0.1112	9	RMETALS	0.1532
*h(q): − 4,4*					
1	RREALESTATE	0.3169	1	RINFORMATIONTECH	0.293
2	RMETALS	0.227	2	RTOURISM	0.2792
3	RINDUSTRIAL	0.2265	3	RINDUSTRIAL	0.2685
4	RCHEMPETPLAS	0.1798	4	RBIST	0.2601
5	RTOURISM	0.1703	5	RFINANCIAL	0.256
6	RINFORMATIONTECH	0.1415	6	RSERVICES	0.2208
7	RFINANCIAL	0.1068	7	RCHEMPETPLAS	0.2169
8	RBIST	0.1057	8	RREALESTATE	0.2060
9	RSERVICES	0.0929	9	RMETALS	0.1307
*h(q): − 3,3*					
1	RREALESTATE	0.3220	1	RINFORMATIONTECH	0.2998
2	RMETALS	0.2320	2	RTOURISM	0.2906
3	RINDUSTRIAL	0.2316	3	RINDUSTRIAL	0.2750
4	RCHEMPETPLAS	0.1769	4	RBIST	0.2645
5	RTOURISM	0.1693	5	RFINANCIAL	0.258
6	RINFORMATIONTECH	0.1139	6	RSERVICES	0.226
7	RFINANCIAL	0.1091	7	RCHEMPETPLAS	0,226
8	RBIST	0.1076	8	RREALESTATE	0.2188
9	RSERVICES	0.0720	9	RMETALS	0.1038
*h(q): − 2,2*					
1	RREALESTATE	0.3277	1	RINFORMATIONTECH	0.3075
2	RMETALS	0.2345	2	RTOURISM	0.3045
3	RINDUSTRIAL	0.2374	3	RINDUSTRIAL	0.2831
4	RCHEMPETPLAS	0.1842	4	RBIST	0.2694
5	RTOURISM	0.1683	5	RFINANCIAL	0,2600
6	RINFORMATIONTECH	0.1283	6	RSERVICES	0.2319
7	RFINANCIAL	0.1150	7	RCHEMPETPLAS	0.2365
8	RBIST	0.1133	8	RREALESTATE	0.2357
9	RSERVICES	0.049	9	RMETALS	0.086
*h(q): − 1,1*					
1	RREALESTATE	0.3323	1	RINFORMATIONTECH	0.3188
2	RMETALS	0.2429	2	RTOURISM	0.3169
3	RINDUSTRIAL	0.2426	3	RINDUSTRIAL	0.2906
4	RCHEMPETPLAS	0.1901	4	RBIST	0.2735
5	RTOURISM	0.1675	5	RFINANCIAL	0.2615
6	RINFORMATIONTECH	0.1217	6	RSERVICES	0.2368
7	RFINANCIAL	0.1194	7	RCHEMPETPLAS	0.2353
8	RBIST	0.1177	8	RREALESTATE	0.2522
9	RSERVICES	0.0488	9	RMETALS	0.0877

**Table 8:** Average of the MF-DFA rankings for the aggregate and sectoral market returns

The GFC			The COVID-19 Outbreak		
Ranking	Sector	MDM	Ranking	Sector	MDM
1	RREALESTATE	0.3223	1	RINFORMATIONTECH	0.3013
2	RMETALS	0.2321	2	RTOURISM	0.2924
3	RINDUSTRIAL	0.2321	3	RINDUSTRIAL	0.2762
4	RCHEMPETPLAS	0.1811	4	RBIST	0.2648
5	RTOURISM	0.1352	5	RFINANCIAL	0.2579
6	RINFORMATIONTECH	0.1338	6	RSERVICES	0.2278
7	RFINANCIAL	0.1155	7	RCHEMPETPLAS	0.2249
8	RBIST	0.1139	8	RREALESTATE	0.222
9	RSERVICES	0.074	9	RMETALS	0.1122

## Conclusion

The primary motivation for this study is to show which sectors have higher efficiency during various crisis periods. Market efficiency is an important indicator of whether or not an investor can profit from a return. In fact, some investments remained significant during the crisis periods. Depending on the motivation, we explore the time-varying market efficiency for sectoral markets using the MF-DFA to understand the features of the market periods, such as structural revolutions throughout the GFC and COVID-19 outbreak. Therefore, this study aims to contribute to the literature on AMH by performing the MF-DFA on aggregate and sectoral markets in Turkey during the GFC and COVID-19 outbreak.

Our results have many intriguing implications for policymakers and investors. First, the findings show that all sectoral markets exhibit persistent behavior and multifractal characteristics, implying that arbitrage opportunities increased during the two crisis periods. The changing Hurst exponents at various time scales validate the AMH, in which arbitrage opportunities vary over time. The persistence levels of the sectoral markets differ between the two crisis periods, and speculative behaviors in all sectors are more prominent due to the features of each crisis, and follow a stronger behavior during the COVID-19 outbreak than during the GFC. This result supports the empirical findings of Choi (2021) and Mensi et al. (2021).

Second, the real estate and information technology sectors had the lowest efficiency during the periods of the GFC and the COVID-19 outbreak This finding is consistent with those of Choi (2021) and Caporale et al. (2021). As a result of the arbitrage opportunities in these markets, investors can generate abnormal returns by employing trading strategies. Their efficiency levels may be developed through increased information flow for greater transparency, more active investment strategies that shift based on the changing efficiency levels, and improved regulatory institutions. However, as an indicator of economic performance, the services and metals sectors are seen to be the most effective during a crisis. In terms of policy stance, an efficient market plays an important role in economic development by facilitating capital formation, resource allocation, and wealth distribution. Changes in market efficiency may also influence investors’ expectations about future economic conditions. The recent financial and economic crises, such as the GFC and COVID-19, reveal how an inefficient stock market can disrupt economic growth. Therefore, our results may serve as a guide for policymakers and regulators in decreasing economic disruptions through revisionary measures. In terms of portfolio management, investors can consider a diversification strategy based on the differences in the current state of efficiency of sectoral markets due to an efficient market with a time-varying structure.

Third, due to the sovereignty of small fluctuations, all sectoral stock returns exhibit multifractality and long memory. During the GFC and COVID-19 outbreak periods, the real estate and industrial sectors have the strongest persistence, whereas the services and metals sectors have the weakest. It can be stated that previous period prices affect the formation of current prices in the real estate and industrial sectors due to their long memory features for the GFC period. In terms of policy, policymakers can enact regulations to increase market forecasting and attract various types of investors to these sectors. However, price changes in the services and metals sectors are not estimated by past price movements because they were the most efficient among the others during the COVID-19 outbreak.

The findings reveal that convenient portfolio allocation, risk diversification, and hedging strategies may provide higher economic development. In other words, ensuring the sustainable development of the economy and sectors and the stock market revolution should be encouraged to maintain a dynamic and sustainable market. Moreover, market mechanism arrangements should be used rationally to decrease the effect of exogenous events on market efficiency. The investor structure in the stock market should be optimized, and the investment value of companies should be increased to provide relative benefits over financial intermediation. If the measures are implemented correctly, they will help boost market confidence, stabilize market sentiment, and encourage long-term improvement in the Turkish stock market.

## Data Availability

The authors confirm that data will be made available on reasonable request.

## Abbreviations

GFC: Global financial crisis
COVID-19: COVID-19 pandemic crisis
MF-DFA: Multifractal detrended fluctuation analysis
EMH: Efficient market hypothesis
AMH: Adaptive market hypothesis
